# Unpacking categorizations in researching GBTIQ+ parents

**DOI:** 10.1177/13634607241228113

**Published:** 2024-01-16

**Authors:** Carole Ammann

**Affiliations:** ETH Zürich, Switzerland

**Keywords:** Fatherhood, parenthood, queer kinship, rainbow-families, same-sex families

## Abstract

In this article, based on anthropological research conducted in the Netherlands and Switzerland, I show the diversity and multi-faceted nature of GBTIQ+ (gay, bisexual, trans, intersex, and queer) parenting. In contrast to recent research on GBTIQ+ parents, which often distinguishes between parents who have children through a (former) heterosexual encounter, adoption, fostering, surrogacy, co-parenting, or trans pregnancy, I deliberately chose not to study just one form of family formation. Drawing on 37 biographical, narrative, and thematic interviews and two group discussions with GBTIQ+ parents, I adopt a processual understanding of parenting that takes into account its fluidity and transformations over the life course. I argue that we should pay attention to how both the unique ways of forming and being a GBTIQ+ family, and common notions of imagining and doing family, intermingle in practice. Furthermore, I stress the importance of taking into account the intersecting differences within the category of GBTIQ+ parents, and accordingly, we should critically analyze which factors are relevant to an individual in a particular time and space.

## Introduction

Jorik was born in the Netherlands. From an early age, he was aware of his same-sex desires and he always imagined himself as a father. When Jorik was in his early thirties, he was contacted by a single woman who was searching for a co-parent. At that time, the relationship between Jorik and Hendrik, his current partner, was on halt and thus, Jorik made the decision to become a single father. Soon after his child was born, Jorik and Hendrik became a couple again. Some months later, a friend of their child’s mother asked the couple whether they wanted to co-parent a second child and they agreed, this time with Hendrik as the biological father. Since then, the two men parented two biologically non-related children in a fifty-fifty care arrangement with two different mothers (interview, April 2021).

Jonathan was born in a West European country and grew up believing that he was a woman who was attracted to men. After his coming out as a trans man and moving to Switzerland, Jonathan and Stefan, his husband at the time, decided to make use of Jonathan’s bodily abilities to become pregnant, “even though I am a man,” as he said. Until 2 years ago, the couple and their two children lived together in what Jonathan labeled as a “traditional nuclear family” with two fathers. Since their separation, Jonathan identified as a single father who took care of his children for half of the week (interviews, September and October 2021).

Juan was born in Latin America. He left his country of origin when he was in his late twenties, only a few months after his child was born. Acknowledging his same-sex desires, Juan was convinced that he and the mother of his child would never be happy together. Once in the Netherlands, Juan fell in love with Hans who had just become a father in a co-parenting arrangement, in which the child lived with the mother and spent every third weekend with him. Juan was in daily contact with his now adult child who remained in his country of origin. Reflecting about being a transnational father, Juan stressed: “I was not physically there, but I was always emotionally present” (interview, November 2020).

Adriaan grew up in a small Dutch city. When he and his partner Patrick desired to become fathers, they registered as foster parents. Six years ago, the couple began to take care of a foster child every third weekend. Four years later, two siblings additionally entered their household on a full-time basis. The siblings met their biological parents, who were separated, as well as their older half-siblings, on a monthly basis (interview, February 2021).

The writing of this article was triggered by several puzzles I encountered while conducting anthropological research for two different, but related projects: one on fatherhood in the Netherlands and one on GBTIQ+ (gay, bisexual, trans, intersex, and queer) parents^1^ in the Netherlands and Switzerland. First, when GBTIQ+ parents are portrayed in the media, we rarely see the different ways they can become parents and the different family constellations they live in. Instead, we see a stereotypical image of the nuclear family with two gay fathers. However, the four portraits above, along with the other data on which this article is based, show that there is not one way of becoming and being a GBTIQ+ parent. GBTIQ+ (intended) parents follow different routes to family formation, and their ways of doing family (Jurczyk, 2014) are multiple and change over time.

Second, I asked myself what gaps in knowledge exist in research on GBTIQ+ parents and how might these be a consequence of the primacy given to practices of conception and family formation in research design, which typically distinguishes between having children through a (former) heterosexual relationship, adoption, fostering, surrogacy, or trans pregnancy (see literature review). Third and more generally, I wondered whether it is always useful to distinguish between heterosexual cis-gender fathers and GBTIQ+ parents in qualitative research on parenting. As GBTIQ+ parents do not fit into the hegemonic family norm consisting of the bourgeois, cis-heterosexual, two-parent family, there are obviously significant differences between the two categories. On a biological level, it is significantly more difficult for GBTIQ+ people to become parents than it is for heterosexual cis-gender men. Typically, the former do not become parents by chance, and thus, their family formation process revolves primarily around the question of how to become parents.

As a result, on an individual level, GBTIQ+ people need to invest a lot of time in gathering the necessary information, considering the different options available to them, and therefore, show more willingness and perseverance in pursuing their desire to become parents. Because of the long path to parenthood, GBTIQ+ intended parents usually think more in advance about what kind of family they want to be and how they want to parent in their everyday lives. On an economic level, they also need to have sufficient financial resources when it comes to surrogacy and, to a lesser extent, adoption (Berkowitz, 2020; Carneiro et al., 2017). Accordingly, the children of GBTIQ+ parents are highly desirable. Most heterosexual cis-gender couples, in contrast, have the privilege of thinking about the “best” time to become parents. Furthermore, GBTIQ+ families do not fit into the dominant cis-heteronormative structures. On a legal level, Dutch and Swiss laws consider the heterosexual nuclear family as the norm (Nay, 2019). As a result, GBTIQ+ families face legal uncertainty and personal vulnerability, for example, due to the long, emotional, and administratively intensive path to legal recognition as parents—which may even be impossible, for example, if a child has more than two social parents.^2^

Fourth, on a social level, parenting in a cis-heteronormative world can be very energy consuming, for example, because GBTIQ+ parents are regularly confronted with highly personal and transgressive questions. Thus, they are in Nebeling Pietersen’s (2018) words doing “affective work,” that is the everyday invisible efforts minorities make to fit into dominant heteronormative structures. However, when it comes to daily parenting, heterosexual cis-gender fathers and GBTIQ+ parents often enjoy similar pleasures and face comparable difficulties (Dahl and Gabb, 2019: 223).

Concerning whether research should generally differentiate between the two categories, the views of the research participants varied. Giacomo, who wished to become a parent through surrogacy, stated, for example, that heterosexual cis-gender fathers and GBTIQ+ parents should not be analyzed together, “because we are a minority group about which society has stereotypical assumptions. We must deal with specific traumas and strictly community related issues that are not present in the heterosexual world” (follow-up interview, February 2022). In contrast, Jonathan, the trans father introduced at the beginning of the article, explained: “Our experience is that when it comes to parenting, typical child-related issues that everyone has in common quickly come to the fore. Actually, the closing of ranks [between GBTIQ+ and heterosexual cis-gender parent families] around children’s issues happens very quickly, while being trans or gay is pushed to the background” (interview, September 2021). Jonathan’s answer mirrors a study by Forenza et al. (2021) of LGB+ adoptive parents, which shows that the parental identity has been used to “normalize” LGB+ identities. Giacomo’s and Jonathan’s responses should not be seen as contradictory but rather as two sides of the same coin. As Heaphy (2018: 162, emphasis in original) illustrates, GBTIQ+ parenting families “can be simultaneously viewed as traditionally conventional *and* post-traditional and non-conventional.”

While this article probably raises more questions than it answers, I propose that we understand parenting and doing family (Jurczyk, 2014) as ongoing processes and thus focus on the ever-changing parenting experiences of GBTIQ+ parents over time and space. We should also consider how different identities and biographies influence a person’s path to parenthood and how processes of family formation influence everyday parenting. We should explore how sexual orientation and gender identity constitute and transform becoming and being parents and, conversely, how parenting impacts on GBTIQ+ identities. Thus, we should pay attention to how both the unique ways of forming and being a GBTIQ+ family, and the general ideas about imagining and doing family (Jurczyk, 2014), intermingle in practice. Furthermore, I stress the importance of taking the intersecting differences within the category of GBTIQ+ parents into consideration, and accordingly, we should critically analyze which factors are relevant for an individual at a specific time and space. Additionally, I suggest that the fluidity of parenting and its transformations over the life course have to be carefully analyzed. In what follows, I will provide a literature review in which I demonstrate recent trends in research on GBTIQ+ parents and then describe my methodological approach. In the empirical section, I use the examples of two GBTIQ+ parents to illustrate my arguments and conclude with a discussion of the main findings.

## Literature review

In Europe, the number, and in particular, the visibility of LGBTIQ+ families, has increased—not least due to recent legal changes around the issues of partnership, marriage, and parenthood, as well as the emergence and legalization of new reproductive technologies (Dahl and Gabb, 2019). The growing number of LGBTIQ+ families is also reflected in the increase in qualitative and quantitative studies on the topic. Five decades ago, research started exploring “alternative” forms of LGBTIQ+ families (Heaphy, 2019: 19), which two decades later have been termed “chosen” families (Weston, 1991). Initially, there was a principal focus on lesbian mothers and their children (Carneiro et al., 2017), but recently, research on GBTIQ+ parents grew (for overviews, see Björklund and Dahl, 2019; Carneiro et al., 2017; Gabb and Allen, 2020; Goldberg and Allen, 2020; Reczek, 2020; Teschlade et al., 2020; Wahlström Henriksson and Goedecke, 2021).

Over the last two decades, there has been a generational shift, which is also evident in my data: whereas the vast majority of older GBTIQ+ parents had children through a heterosexual encounter, younger GBTIQ+ people are more likely to use surrogacy, adoption, fostering, or become parents through various co-parenting arrangements (Patterson and Tornello, 2011; Tasker and Lavender-Stott, 2020; Weeks et al., 2001). Interestingly, however, there are not many recent studies focusing on GBTIQ+ parents following the dissolution of a heterosexual relationship (except for Clarke and Earley, 2021; Tasker and Lavender-Stott, 2020). This may be related to a tendency in academia to focus on the novel and overlook the ordinary (Dahl and Gabb, 2019: 215). In contrast, gay couples who have become parents through surrogacy seem to be *the* hot topic in current GBTIQ+ parenting research (e.g., Berkowitz, 2020; Dempsey, 2014; Knoll and Moreno, 2020; Lustenberger, 2017; Majumdar, 2016; Murphy, 2013; Nebeling Petersen, 2018; Smietana, 2018; Tsfati et al., 2021; Wei, 2021).

Less researched groups include GBTIQ+ people who foster children and GBTIQ+ stepfamilies (but see Bergeson et al., 2020; Bermea et al., 2019; Riggs, 2020; Sagert, 2021), as well as GBTIQ+ adoptive parents (but see Costa and Tasker, 2018; Farr and Vázquez, 2020; Forenza et al., 2021; Messina and D’Amore, 2018).^3^ Furthermore, there is almost no literature on GBTIQ+ co-parenting (but see Bos, 2010; Liu, 2013) nor on GBTIQ+ polyamorous parent families (but see Klesse et al., 2022; Pain, 2020), both of which challenge the normative ideal of the GBTIQ+ two-parent family. Furthermore, there are still significant research gaps regarding bisexual parents, single GBTIQ+ parents, GBTIQ+ donor parents, and gender-neutral, gender-fluid, or gender-diverse parents (Reczek, 2020; Riggs et al., 2016). In contrast, the literature on transgender parenting has grown lately (e.g., Downing, 2013; Hafford-Letchfield et al., 2019; Pfeffer and Jones, 2020; Riggs et al., 2021; Stoll, 2020). Overall, recent research on GBTIQ+ parents tends to analyze a specific category, usually along the lines of how they became parents, or to a lesser extent, a specific letter of the GBTIQ+ acronym. Furthermore, most research focuses on white, middle- or upper class, monogamous, cis-gender two-parent families (Carone et al., 2021; Dahl and Gabb, 2019; Reczek, 2020).

### Notes on data collection

While the writing of this article is informed by all the data collected in the two anthropological research projects, here, I draw only on 37 biographical, narrative, and thematic interviews and two group discussions I conducted with research participants who identified as GBTIQ+ (intended) parents and not as female. Data collection took place between April 2020 and June 2022 in Switzerland and the Netherlands.^4^ I deliberately chose not to research just one form of family formation. Consequently, the sample consists of people from various backgrounds living in different family constellations. They were aged between 30 and mid-60 and had become parents between the ages of 23 and 48. They had between 0 and 3 children, some were biologically related and some were not. A total of 13 parents had their children in a co-parenting arrangement, 13 became a parent through a heterosexual relationship, 10 used surrogacy, 3 carried their children themselves—either before or after their coming-out as trans—1 was a foster parent, and 1 person hoped to adopt in the near future. All but five of the participants were highly educated and six described themselves as racialized “others” within the local context. A total of 15 grew up in the Netherlands, 13 in Switzerland, 13 in other European countries, and 4 in the Americas. With the exception of three transgender fathers and one trans, non-binary person, all participants identified as cis-gender. The amount of time the parents spent with their children varied widely: while one man described himself as a donor plus father, others had their children full time.

The interviews were conducted either in Dutch, English, German, Swiss German, French, or Spanish, depending on the participants’ preference. Depending on the COVID-19 regulations in place, the research participants could choose between meeting personally or an online interview. For the face-to-face interview, the participants could choose between a walking interview or a sitting interview in a place of their choice, such as in a park, a coffee, or at their own home.^5^ I had a second interview with seven of the parents and also followed-up with many of them through email exchanges to keep abreast of recent changes—not least in relation to the impact of COVID-19 on their families. All interviews were audio-recorded with consent and later transcribed. I coded them according to the principles of grounded theory (Charmaz, 2006), using the MAXQDA software (Kuckartz and Rädiker, 2019). I identified recurring themes through initial codes, which were then developed into focused codes as I read the data more closely. I gave each participant a pseudonym to ensure their anonymity. In addition to the interviews and group discussions, I collected government documents, (online) media articles, blog posts, newsletters, watched documentaries and TV series, listened to podcasts, and followed parenting groups on Facebook in order to analyze ongoing discussions about (LGBTIQ+) parenting in Switzerland and the Netherlands. While all the above data has informed this article, as is typical in social anthropological writing, I will focus on a few cases here to make my points.

The impact of researchers’ positionalities and the question of “insider” versus “outsider” when conducting research “at home” or “away” is a prominent theme in social anthropology and related disciplines (e.g., Caputo, 2003; Hannerz, 2006; Mughal, 2015; Sultana, 2007). The positionalities of both researchers and research participants are always multidimensional (Dery, 2020) and intersectional (Lozano-Neira and Marchbank, 2016). Even when research participants represent a particular group, such as GBTIQ+ parents in this case, they are never homogeneous but diverse in terms of, for example, family constellation, age, educational background, origin, and/or gender identity. Gaining access and trust depends on multiple factors and may or may not be facilitated by homogeneity on one social dimension (Ammann, 2021; Merriam et al., 2001; Valiquette et al., 2021). Both straight and queer researchers have reflected on how their sexuality—and its revelation or hiding—has influenced their research with LGBTIQ+ people (e.g., Adeagbo, 2018; Dagkouli-Kyriakoglou, 2021; Maulod, 2020; Rooke, 2009).

The author is a white, highly educated, cis-gender woman in her early forties who grew up in Switzerland and is not part of the LGBTIQ+ community. Together with her cis-gender male partner and their two children, she spent a year and a half in the Netherlands. The author shared some characteristics with the research participants, particularly her experience of parenting and (for the majority of them) her class background. In contrast, as a white, heterosexual, cis-gender woman, she had a different gender identity and did not share any discriminatory experiences of being confronted with racism, homophobia, and/or transphobia. Being a member of the LGBTIQ+ community would certainly have helped to gain a deeper and more embodied understanding of certain issues, such as coming-out stories. Overall, however, the research participants were happy to share their experiences and were open about them. During the writing process, I repeatedly discussed the content of the manuscript with (queer) colleagues who specialize in (queer) gender research (see acknowledgments). The study on GBTIQ+ parenting in the Netherlands received ethical approval from the Amsterdam Institute for Social Science Research (AISSR) at the University of Amsterdam. The University of Lucerne, where I was based during data collection in Switzerland, did not have an ethic committee at the time.

### Processual understanding of GBTIQ+ parenting: ordinary changes and major disruptions

Angel grew up in a Caribbean country and became the father of Julio when he was in his mid-twenties. His relationship with Evelyn, Julio’s mother, deteriorated and they separated when Julio was 1 year old. In his country of origin, Angel found it difficult to come-out and have a same-sex relationship, and he feared that Julio would also face discrimination. When Angel moved to the Netherlands, he wanted to take Julio with him, to offer him a “better” future, but Evelyn refused. In the years that followed, Angel had little contact with Julio because there was no internet connection in his country of origin and the phone calls were extremely expensive. He was only able to speak to Julio for a few minutes each month. When Angel traveled back for holidays, finally seeing his child again felt like meeting a stranger, which he found heart-breaking.

But things changed when Evelyn and her new partner decided to move to the US. Angel again asked if Julio could come to the Netherlands, and this time, Evelyn agreed. So, at the age of 13, Julio moved in with Angel and his husband Dario. Looking back, Angel remembered the many years without having Julio around as difficult. Angel wanted to be a different father to Julio than his own father had been to him. He was happy with their current relationship, and Dario was like a second father to Julio. In the autumn of 2021, their family situation changed once again, as Angel and Dario had both fallen in love with another man who came to live with them, expanding their household to three adults and one teenager (interviews, December 2020 and September 2021, informal conversation, November 2021).

Angel’s story shows one of the most important points about parenting, namely, that it is subject to constant change and is therefore “embedded in temporal, spatial, and cultural specificities” (Wahlström Henriksson, 2020: 326). For many years, Angel was a transnational father who only had sporadic telephone contact with Julio. As a result, the two were hardly able to develop a father–child relationship. When they finally met again, they had a lot of catching up to do. Julio, for example, began to ask questions about Angel’s same-sex relationship. After more years of being a father-at-a-distance, Angel suddenly found himself being a full-time father. Although he had Dario’s support, Angel felt the weight of this new responsibility. The first few months were dominated by questions about Julio’s adjustment to the new environment. As Julio grew older and his relationship with his fathers changed, other issues became important, such as whether they should allow him to have a part-time job alongside school. Then, for a period of time, Julio spent a lot of time with his new friends but did not want to introduce them to Angel and Dario. Angel suspected that it was because he had two fathers.

Angel’s story highlights that transformations are an integral part of family life: new children are born, children grow up, job situations change, families move to another place, health conditions alter, couples separate, and new relationships form. For Angel, the separation from Evelyn and his coming-out led to less contact with Julio—partly because Evelyn had difficulty accepting his homosexuality and prevented Julio from seeing his son—and later due to being a transnational father. We should therefore consider GBTIQ+ parenting as fluid and processual, much like heterosexual cis-gender parenting, and thus take into account the many changes over the life course (Aeby and Gauthier, 2021; Dahl and Gabb, 2019). Research that only looks at the becoming of GBTIQ+ parents and the processes of family formation is unable to capture these transformations. However, capturing this long-term dimension is often a challenge for qualitative research due to the lack of available money and time in current neoliberal academia.

The separation of, or the dissension between, GBTIQ+ parents is a critical moment that has a significant impact on parenting, particularly if it is accompanied by a rupture of the child–parent relationship. While break-ups are also often challenging for heterosexual cis-gender couples, a separation and/or a dissension presents additional difficulties for GBTIQ+ parented families (Carmeli, 2020; Gahan, 2018; Tasker and Lavender-Stott, 2020). First, as Angel’s example and the discussions I witnessed at meetings of two different “gay dads”-groups showed, GBTIQ+ parents in heterosexual relationships fear losing their children if they disclose their sexuality or gender identity.

Second, in GBTIQ+ co-parenting families with more than two social parents, the legally unrecognized parents are in very vulnerable positions. These vulnerabilities do not only manifest themselves in major ruptures such as break-ups; they can also be subtle as Dieter’s example shows. Dieter co-parented three children with a lesbian couple. He was the biological father of the first two children, while Dieter’s partner Florian was the biological father of the third one. Florian had no care mandate but spent time with Dieter and the children from time to time. Legally, the two mothers were the children’s parents, and they had not written an informal agreement about Dieter’s and Florian’s rights and responsibilities as fathers before the birth of his oldest child. While Dieter was quite involved when his first child was born, the mothers did not want him to take the second child home until it was 2 years old, and he was even less involved with the third child. Thus, the amount of time Dieter spent with his children varied according to his relationship with, and the demands of, the two mothers. As the biological mother could legally deny him access to his children altogether, Dieter felt in a weak position (interview, February 2022).

Third, GBTIQ+ parents often feel the pressure to portray themselves as “good” parents and their children as doing well (Carneiro et al., 2017: 9). Thus, a separation disrupts the image of the “happy queer family” (Gahan, 2018; Nay, 2015) that is often promoted in the (progressive) media or by LGBTIQ+ organizations. Frédéric is a case in point. He and his former partner Christophe separated when their child’s surrogate was pregnant. At the time of the interview, they were still living together and caring for their one-and-a-half-year-old child. Frédéric stressed that their separation caused incomprehension among friends and families who had just begun to accept their decision to become gay parents through surrogacy. “Suddenly, we were out of the pattern of what was expected of us. […] I feel like we have to work extra hard to show that our child is doing well” (interview, January 2022).

### Intersectional understanding of GBTIQ+ parenting: multiple and sometimes shifting identities

Raj is of mixed Dutch, Asian, and Caribbean descent. They grew up in a very religious evangelical family in a Dutch city. When they were born, Raj’s parents thought they were a girl and raised them accordingly. Raj was an active member of their religious community. In line with their religious beliefs, Raj was convinced that having same-sex desires and being trans were demonic and thus, they prayed to be “cured,” but praying did not help. Raj was in their early twenties when they came out and began transitioning. At the time of the interview, they identified as gay and non-binary. After their coming-out, the church expelled Raj and they completely changed their life as a result. Raj moved to another city and looked for new friends, whom they found mostly within the queer community.

On the subject of parenting, Raj said: “I grew up with the idea that I would never have children, because I didn’t want to be a mother. I knew this very clearly from a very young age. […] But things changed when I held my sister’s one-day old baby in my arms. Then I thought: I do want children, but I don’t want to be a mother.” From that moment on, Raj was convinced that they would become a parent one day, even if they remained single. When Raj fell in love with their current husband Gabriel, a cis-gender man, they both wanted to be parents. But their journey to parenthood was not straightforward. Stopping the testosterone led to severe mood swings. Finally, and after months of vain hope and disappointment, Raj became pregnant with medical support. The couple gave their child a gender-neutral name and planned to raise them in a gender-neutral way, until they could clearly communicate their gender identity (interviews, March and December 2021).

Raj’s story illustrates the need to take an intersectional approach when researching GBTIQ+ parenting. Raj’s religious background, for example, prevented them from accepting their trans identity and same-sex desires. Research confirms that belonging to a religious community that does not affirm same-sex relationships can be detrimental to health and a barrier to coming out (Tasker and Lavender-Stott, 2020: 5). Raj actively fought against their feelings by praying “to be changed.” After their coming out as trans and non-binary, Raj changed both their place of living and their social environment. Those of their family who were still active members of Raj’s former religious community did “not believe in trans,” as Raj put it. Raj’s mother, for example, still referred to them by their dead (birth) name and thought of Raj as her daughter. At the same time, both parents were supportive of the couple’s journey to parenthood. The inability to conceive without medical assistance had further impact on Raj, not least because it prolonged the time that they were unable to take testosterone which had negative impacts on their emotional stability and bodily feeling. For Raj, their Asian background was also of major importance because, as they explained, historically there was more room for queer identities than within the Dutch cis-heteronormative environment. Being racialized within the local context, Raj belonged to a further minority. Furthermore, even within their community of trans people and trans men, not everyone understood why Raj wanted to carry a baby themself.

Raj’s example highlights that the intersectional identities of sexuality, ethnicity, religion, and gender identity can crucially influence GBTIQ+ parenting (Carneiro et al., 2017: 10, Dahl and Gabb, 2019). Other dimensions of a person’s identities and biographies also shape parenting, such as class, age, age of becoming a parent, one’s health, (dis)ability, migration, citizenship, working sector, level of employment, experiences of discrimination, and family constellations. Importantly, however, we should not view such intersecting identities as static but rather in relation to time and space. Because parenting is a process, some positionalities, such as Raj’s identification as non-binary which influenced their desire to raise their child in a gender-neutral way, may become more salient, while others, such as Raj’s religiosity, may become less so.

Another example is Piet, who, as a gay man, thought that the only way to become a parent was through adoption, which seemed to be very complicated. However, when Piet met a man who was in the process of becoming a father in a co-parenting constellation, new possibilities emerged and becoming a parent became “thinkable” (Smietana, 2022). At the time of the interview, Piet was a single man who co-parented his child with its mother. He told me that he had underestimated the challenges of being responsible for a young child alone for half of the week. This highlights that while same-sex desires shaped Piet’s journey to parenthood, being a single father had a much greater impact on his parenting once the baby was born (interviews December 2020 and January 2022). Similarly, Forenza et al. (2021: 18) argue that “While experiences as ‘lesbian’ or ‘gay’ prospectively informed participant conceptions of family, the paramount identity of ‘parent’ retrospectively usurped all other collective identities.”

Levi, a single gay father, became a parent at an advanced age. He lamented: “I think I am too old for this project, but I didn’t know that before. I didn’t know that I had physical limitations. […] So unfortunately, I am not a fit father” (interview, June 2020). For Levi, therefore, it was his advanced age when he became a parent, rather than his sexuality, that was the most influential factor in his parenting of two young children. All of these examples illustrate the important point made by Dahl and Gabb (2019: 218) when they argue that the various “(contextual) factors […], experiences and circumstances that differentiate LGBTQ parent families from each other […] are, arguably, equally or even far more telling of what shapes LGBTQ parenthood.” It is therefore important to recognize the diverse and sometimes changing identities of GBTIQ+ parents, as well as the great diversity within GBTIQ+ families.

People like Raj, whose identities consist of additional minority positions, are underrepresented in GBTIQ+ parenting research (Carone et al., 2021; Dahl and Gabb, 2019; Reczek, 2020). One reason for this is that researchers have not been sufficiently successful in accessing the existing GBTIQ+ parents beyond white, cis-gender, middle-class, gay men, as scholars often conduct their research by tapping into GBTIQ+ networks (Dahl and Gabb, 2019: 216). Indeed, the systematic review on gay and bisexual fatherhood by Carneiro et al. (2017: 3) found that the vast majority of the studies, namely, 91%, collected data in this way. And as Dahl and Gabb (2019: 216) point out, these arenas “are heavily dominated by articulate, rights-aware and relatively ‘privileged’ LGBTQ people.” This is not surprising given the current neoliberal academic environment, where scholars lack the time and resources necessary to conduct “slow” research (Mountz et al., 2015; Schwiter and Vorbrugg, 2021) and, thus, access hard-to-reach people (Dahl and Gabb, 2019: 215–217). While the data presented here reflects some diversity in terms of gender identity, class, race, origin, path to parenthood, and family constellation, I was unfortunately unable to better include further minorities within the sexual and gender minorities in the Netherlands and Switzerland.

The second reason for the underrepresentation of minorities in GBTIQ+ parenting research is, as Heaphy (2019: 23) rightly argues, that “the possibilities […] of LGBQ in terms of family, kinship and intimate citizenship have radically changed for some but remain unchanged for others.” Often, those for whom the possibilities have not changed due “to problems of access and opportunity” (Donovan and Wilson, 2005: 133), are marginalized GBTIQ+ people. They face even greater challenges in becoming parents than white, middle class, cis-gender gay men, especially if it is not through a (former) heterosexual encounter. Many factors support the pathway to parenthood, such as ability, being in good health, having secure housing and employment, support from a partner, family, and friends, citizenship rights, and sufficient financial resources. Thus, Carneiro et al. (2017: 10) note that “(non-white) gay and bisexual men with lower income and educational level may not in fact be able to undertake these routes to parenthood.”

### By way of concluding

I began this article by describing some of the puzzles I have faced in conducting research on fatherhood and GBTIQ+ parenting. First, I considered whether it is useful to generally make a distinction in parenting research between heterosexual cis-gender fathers and GBTIQ+ parents in parenting research. Pralat (2021: 276), for example, suggests to “think of people in terms of their ‘reproductive orientations’ rather than sexual identities.” Other scholars suggest that in everyday life, a parent’s sexuality or gender identity is of secondary importance, as all parents face the ordinary joys and difficulties of family life (Dahl and Gabb, 2019: 223). As such, being a parent is normalizing for GBTQ+ people, for example, in their relationship with their families of origin (Forenza et al., 2021).

However, there are still many instances where GBTIQ+ parents are directly or indirectly confronted with homophobia and/or transphobia and, more generally, cis-heteronormative structures on the social, political, economic, and legal levels. As the ordinary family lives of GBTIQ+ parents simultaneously disrupt and reinforce normative notions of the cis-heterosexual, two-parent family norm (Heaphy, 2018), I argue that research should look closely at the similarities and differences between and among GBTIQ+ parents and heterosexual cis-gender fathers and to critically reflect on when and why it makes sense to differentiate between the two groups.

Second, Angel’s, Raj’s, and the other research participants’ stories illustrate the great diversity of GBTIQ+ people’s lives before and after becoming parents. As I illustrated in the review of recent literature considered in this paper, scholars have often distinguished between people who became parents in the context of a (former) heterosexual relationship, adoption, fostering, trans pregnancy, co-parenting, or through surrogacy. However, sampling along these lines can, on the one hand, miss the differences within a group because there is no single experience of, for example, being a GBTIQ+ adoptive parent. On the other hand, how GBTIQ+ persons become parents is not the only criterion that determines their parenting. Other and sometimes changing aspects of the parents’ identities, such as their age, religion, class, ethnicity, gender identity, and (dis)ability among others, also impact their parenting. Therefore, an intersectional approach that is sensitive to space and time is crucial when researching GBTIQ+ parents.

Third, and most importantly, we should not think of parenting as fixed but as fluid, dynamic, and constantly changing. Men are not simply “involved” or “new” fathers if they participate more in their children’s upbringing than their own fathers did. On the contrary, parenting is a constant process of transforming, change, and shifting, and thus, of becoming. People do not have a family but are doing family (Jurczyk, 2014), through routines and rituals. Future research should therefore pay close attention to the shifting and intersecting identities of GBTIQ+ parents. This would then allow us to analyze how these factors affect parenting over time and space as children and parents grow older, and as family situations and structural factors change. In summary, I advocate for a processual understanding of GBTIQ+ parenting that pays attention to the experiences and changes that occur across the life course. This, I argue, will help us to move beyond dichotomies, such as that between heterosexual cis-gender fathers and GBTIQ+ parents, or the process of grouping GBTIQ+ parents solely along the lines of family formation.

## Notes

1. While the B for bisexual is included in this acronym, I explicitly focus on persons who do not identify as women.

2. At the time of writing (2022), the topics of multi-parenting and surrogacy were being discussed within the Dutch parliament (see, e.g., https://www.tweedekamer.nl/downloads/document?id=9b427f6b-7036-4fd3-8489-9a73a277875c; https://www.tweedekamer.nl/kamerstukken/stemmingsuitslagen/detail?id=2022P12083).

3. While adoption is generally declining, many gay couples still wish to adopt a child, even though they face numerous obstacles throughout the process (Forenza et al., 2021).

4. Due to the lack of statistical data on GBTIQ+ parents in Switzerland and the Netherlands, we know very little about their number, their background, and how they became parents (but see Dachverband Regenbogenfamilien Schweiz, 2017; Felten and Rothuizen, 2014).

5. I reflected on the differences between the different forms of interview and the advantages of walking interviews elsewhere (Ammann, 2021).
